# Association Between NT-proBNP and Prolonged Length of Stay in Hospital Among Preterm Infants Born at 28–31 Weeks' Gestation

**DOI:** 10.3389/fped.2021.783900

**Published:** 2022-01-24

**Authors:** Jian Ge, Chenghan Luo, Mengyuan Lei, Zanyang Shi, Xinru Cheng, Min Zhao, Yanting Zhao, Min Song, Wenqian Ding, Mengmeng Wang, Wenjun Cao, Fengxia Mao, Suge Han, Qianya Xu, Junbo Wang, Haoqi Qin, Li Wang, Qian Zhang

**Affiliations:** ^1^The First Affiliated Hospital of Zhengzhou University, Zhengzhou, China; ^2^Henan High Risk Newborn Clinical Treatment and Follow-Up Center, Zhengzhou, China; ^3^Zhengzhou Key Laboratory for Prevention and Control of Developmental Disorders, Zhengzhou, China

**Keywords:** N-terminal pro-brain natriuretic peptide, preterm infants, length of stay, cardiac function, duration of hospitalization

## Abstract

**Objective:**

In the early life of preterm infants, the relationship between heart function and length of hospitalization is unclear. This study aims to examine the association between serum NT-proBNP level on the 7th day (NT-proBNP7) after birth and length of hospitalization among preterm infants.

**Methods:**

A retrospective cohort study was conducted. Patients included 709 preterm infants born at 28–31 weeks' gestational age (GA) admitted to the NICU of the First Affiliated Hospital of Zhengzhou University between December 20, 2016, to April 31, 2021. Main outcome: Late discharge (postmenstrual age at discharge was in the fourth quartile (highest) among infants born at the same GA). Exposure factor: NT-proBNP7.

**Results:**

We observed increased prevalence ratios for late discharge among the tertile of logarithm of NT-proBNP7 level (LnNT-proBNP7) which was positive. Compared with the lowest tertile, infants in the highest tertile of LnNT-proBNP7 had an 8.4-fold increased probability of late discharge, and the results were consistent for the subgroups. Next, a non-linear (S-shaped) relationship between LnNT-proBNP7 and late discharge was observed, whose turning points were 7.5 and 9. The effect sizes and the confidence intervals on the left of the first turning point, between two turning points and on the right of the second turning point, were 0.6 (95% CI, 0.2–1.6), 5.0 (95% CI, 2.4–10.6), and 1.1 (95% CI, 0.2–6.1), respectively. In addition, the prevalence of BPD, NEC, nosocomial infection, or any of them was highest in the group of LnNT-proBNP7 ≥ 9, lowest in the group of LnNT-proBNP7 < 7.5.

**Conclusion:**

Higher NT-proBNP7 levels were associated with longer hospitalization. The relationship between LnNT-proBNP7 and late discharge was S-shaped. LnNT-proBNP7 was positively related with late discharge when LnNT-proBNP7 was between 7.5 and 9.

## Introduction

With advances in perinatal medicine, the mortality of very preterm infants has declined in most regions of the world (1, 2). However, prolonged lengths of stay (PLS) in the hospital of preterm infants cause substantial burden on family economic status and health systems (3). Additionally, PLS in hospitals increase the risk of readmissions of infants and are also detrimental to the mental health of parents (4, 5). Therefore, it is necessary to conduct studies on PLS in infants.

There are large differences in the length of hospital stay of very preterm infants among different regions (6). In general, infants with necrotizing enterocolitis (NEC), bronchopulmonary dysplasia (BPD), and nosocomial infection have a longer length of stay in the hospital (7–9).

N-terminal pro b-type natriuretic peptide (NT-proBNP) is an inactive amino acid fragment of the brain natriuretic peptide (BNP) that is released from cardiomyocytes in response to pressure or volume overload, promoting vasodilation and natriuresis (10). NT-proBNP level is associated with impaired cardiac function among children and infants and influenced by kidney function (11–13). In adults, NT-proBNP has been extensively studied in many domains including cardiac diseases, diabetes, and rheumatoid arthritis (10, 14). Several studies found that NT-proBNP was associated with the hospital stay of adult patients with heart diseases (15, 16). However, the relationship between NT-proBNP and the length of hospital stay among infants has not been reported.

For preterm infants, impaired cardiac function may increase the risk of poor growth, feeding difficulty, and weaning failure (17, 18), which could lead to a prolonged length of hospital stay. Recently, studies found that among infants, serum NT-proBNP level in the first week after birth was associated BPD and NEC (19, 20), which are important causes of PLS. Therefore, we speculated that serum NT-proBNP levels in the first week after birth are associated with neonatal intensive care unit (NICU) length of stay among very preterm infants. To verify this hypothesis, we conducted a comprehensive and systematic study in a population of Chinese very preterm infants.

## Methods

### Design and Study Participants

We conducted a retrospective cohort study of very preterm infants born between 28 weeks and 31 weeks plus 6 days of gestational age (GA) and admitted to the Neonatal Intensive Care Unit of the First Affiliated Hospital of Zhengzhou University on their day of birth from December 20, 2016, to April 30, 2021. Infants who had a major congenital anomaly, died during hospitalization, were transferred from another hospital, or were discharged from the hospital without doctor's consent were excluded from the study. This study was approved by the respective research ethics boards of the First Affiliated Hospital of Zhengzhou University, and the written informed consent from the parents/guardians was obtained.

### Outcomes and Variables

The main outcome of this study was late discharge (postmenstrual age at discharge was in the fourth quartile (highest) among infants born at the same GA). In supplementary analyses, we also analyzed total costs during hospitalization and the major neonatal morbidities that may prolong the length of hospital stay, including BPD [defined as oxygen dependency at 36 weeks' corrected GA (21)], NEC [stage 2 or higher NEC according to the criteria of Bell et al. (22)], and nosocomial infection [defined as meningitis or culture-positive sepsis (23)].

The exposure of interest was the level of NT-proBNP at day 7 (NT-proBNP7) of life. To characterize the cohort and to assess potential confounders, both maternal variables and infant variables were collected. Maternal variables included maternal age, cesarean birth, antenatal steroid use, embryo transfer, rupture of membranes for more than 24 h, and hypertension during pregnancy; infant variables included gender, GA, birth weight, small for GA (SGA), Apgar scores at 5 min, respiratory distress syndrome (RDS), NEC in the first week, mechanical ventilation in the first week, NT-proBNP and creatinine level in plasma, and hemodynamically significant patent ductus arteriosus [HSPDA, defined as a ductal diameter of 1.5 mm or larger (24)] at day 7.

### NT-proBNP Assays

Plasma NT-proBNP and creatine levels were measured routinely on day 7 of life, and heart ultrasound was performed on the same day. The Elecsys proBNP II assay was used to measure NT-proBNP on the Roche Modular Analytics E170 analyzer. The measuring range was 5–35,000 pg/ml or 0.6–4,130 pmol/l.

### Statistical Analysis

Considering differences in regions and medical resources, the specific length of hospital stay for very preterm infants may vary in different neonatal intensive care units. Therefore, we converted length of stay in hospital, a continuous variable, to late discharge, a categorical variable. The distribution of serum levels of NT-proBNP at day 7 was strongly skewed left, so it was transformed to the Ln scale (LnNT-proBNP7) for analysis.

The baseline characteristics of the study population were summarized using descriptive statistical methods. To examine the association between population characteristics and LnNT-proBNP7 and late discharge, population characteristics were compared using the χ^2^-test for categorical variables and analysis of variance (F test) for continuous variables.

Then, we explored the dose–response relationship between LnNT-proBNP7 and late discharge and found an S-shaped relationship between them. After determining the turning points of the smoothing plot, we examined the threshold and saturation effects by using a two-piecewise linear regression model. Next, to explain why elevated NT-proBNP level increased the risk of late discharge, we divided the infants into three groups according to the turning points of the curve and compared the prevalence of major morbidities among the three groups.

We selected confounders on the basis of their associations with the outcomes of interest or a change in effect of >10%. Adjustments were made for birth weight, GA, SGA, mechanical ventilation in the first week, HSPDA at day 7, creatine level in plasma at day 7, and hypertension during pregnancy. Missing values were multiply imputed using the “chained equations” method in the R MI procedure (25).

All statistical analyses were conducted using R version 3.6.3 (R Foundation for Statistical Computing, https://www.r-project.org), and a 2-sided *p*-value of 0.05 was used to determine the statistical significance.

## Results

### Participant Characteristics and Univariate Analysis

A total of 800 preterm infants born from 28 to 31 weeks' gestation were admitted on their day of birth to NICU of the First Affiliated Hospital of Zhengzhou University from December 20, 2016, to April 30, 2021, of which 91 infants were excluded from this study (Supplementary Figure 1). The remaining 709 [382 males (53.9%) and 327 females (46.1%)] infants were included in the analysis, and their characteristics are described in Table 1.

**Table 1:** Characteristics of 709 infants.

**Characteristic**	**All patients**
**Infant characteristic**	
Female sex, *n* (%)	327 (46.1%)
**Gestational age, wk**	
28–28^+6^, *n* (%)	84 (11.8%)
29–29^+6^, *n* (%)	156 (22.0%)
30–30^+6^, *n* (%)	194 (27.4%)
31–31^+6^, *n* (%)	275 (38.8%)
Birth weight, g	1,387.7 ± 296.5
SGA, *n* (%)	178 (25.1%)
Apgar score <7 at 5 min	35 (5.0%)
Respiratory distress syndrome, *n* (%)	640 (90.3%)
LnNT-proBNP7	7.7 ± 0.9
Creatine level at day 7, μmol/L	51.4 ± 26.4
HSPDA at day 7	67 (9.8%)
Mechanical ventilation in the first week, *n* (%)	209 (29.5%)
NEC in the first week, *n* (%)	21 (3.0%)
**Maternal characteristic**	
Maternal age >35 y, *n* (%)	131 (18.5%)
Hypertension during pregnancy, *n* (%)	290 (40.9%)
Cesarean birth, *n* (%)	567 (80.1%)
Embryo transfer, *n* (%)	105 (14.8%)
Rupture of membranes ≥24 h, *n* (%)	136 (19.2%)
Antenatal steroid use, *n* (%)	228 (32.2%)

*SGA, small for gestational age; LnNT-proBNP7, NT-proBNP level at day 7 after logarithmic conversion; HSPDA, hemodynamically significant patent ductus arteriosus; NEC, necrotizing enterocolitis*.

*Data are means ± SD, or %*.

In this study, for infants born at 28, 29, 30, and 31 completed week's estimated gestational age (EGA), the postmenstrual ages (PMAs) at discharge were between 238 and 312, 233 and 319, 227 and 319, and 230 and 344 days, respectively. For infants born at 28, 29, 30, and 31 completed week's EGA, the PMAs at late discharge were above 264, 265, 260, and 259 days, respectively (Supplementary Table 1).

The univariate analysis indicated that LnNT-proBNP7 appeared higher among infants that are SGA, and infants with HSPDA at day 7, and infants who had higher creatine level in plasma at day 7 and history of mechanical ventilation. Birth weight and GA were negatively associated with LnNT-proBNP7 (Supplementary Table 2). As is shown in Supplementary Table 3, birth weight, SGA, LnNT-proBNP7, hypertension during pregnancy, and ventilator use were associated with late discharge.

### Relationship Between LnNT-proBNP7 and Late Discharge

In this study, increased prevalence ratios for late discharge among the tertile of LnNT-proBNP7 were positive (p for trend < 0.05). Compared with the lowest tertile, infants in the highest tertile had an 8.4-fold (95% CI, 4.8–14.9) increased probability of late discharge. In subgroup analysis, the association between LnNT-proBNP7 and late discharge was consistent in the following subgroups: gender, GA, SGA, embryo transfer, maternal age, mode of delivery, maternal with gestational hypertension, mechanical ventilation in the first week (all *p* for trend < 0.01 and all *p* for interaction > 0.05) (Table 2).

**Table 2:** The relationship between LnNT-proBNP7 and late discharge and subgroup analysis.

**Group**	***N***	**Adjusted odds ratio (95% CI)**			***p-*** **value for trend**	***p-*** **value for interaction**
		**LnNT-proBNP7 tertile**				
		**T1**	**T2**	**T3**		
All infants	703	1.0 (reference)	1.4 (0.8, 2.4)	8.4 (4.8, 14.9)	<0.001	
Gender						0.6618
Female	325	1.0 (reference)	1.6 (0.7, 3.8)	10.0 (4.1, 24.7)	<0.001	
Male	378	1.0 (reference)	1.3 (0.6, 2.8)	7.7 (3.6, 16.6)	<0.001	
Gestational age, weeks						0.5549
28–28^+6^	84	1.0 (reference)	0.4 (0.0, 7.8)	8.2 (0.9, 76.3)	0.004	
29–29^+6^	155	1.0 (reference)	2.0 (0.5, 8.0)	7.3 (2.0, 26.7)	<0.001	
30–30^+6^	193	1.0 (reference)	1.2 (0.5, 2.8)	17.9 (6.5, 48.9)	<0.001	
31–31^+6^	271	1.0 (reference)	1.3 (0.7, 2.4)	9.6 (5.3, 17.3)	<0.001	
Small for gestational age						0.1023
Yes	176	1.0 (reference)	1.2 (0.5, 2.9)	4.8 (1.8, 13.0)	<0.001	
No	527	1.0 (reference)	1.4 (0.6, 3.4)	15.7 (7.0, 35.4)	<0.001	
Embryo transfer						0.08
Yes	105	1.0 (reference)	0.3 (0.0, 2.0)	16.1 (2.1, 121.6)	0.002	
No	598	1.0 (reference)	1.5 (0.8, 2.8)	10.0 (5.2, 19.0)	<0.001	
Maternal age, years						0.7470
≤ 35	571	1.0 (reference)	1.2 (0.7, 2.4)	9.5 (5.0, 18.1)	<0.001	
>35	131	1.0 (reference)	1.6 (0.3, 8.3)	17.0 (3.1, 91.5)	<0.001	
Mode of delivery						0.3443
Vaginal delivery	140	1.0 (reference)	1.2 (0.2, 6.3)	22.1 (5.0, 96.8)	<0.001	
Cesarean section	562	1.0 (reference)	1.5 (0.8, 2.8)	9.1 (4.6, 17.9)	<0.001	
Maternal with gestational hypertension						0.5704
Yes	287	1.0 (reference)	1.2 (0.6, 2.7)	7.8 (3.2, 18.9)	<0.001	
No	416	1.0 (reference)	1.4 (0.6, 3.5)	13.9 (5.8, 32.9)	<0.001	
Mechanical ventilation history						0.3624
Yes	495	1.0 (reference)	1.6 (0.5, 5.3)	6.7 (2.2, 20.0)	<0.001	
No	208	1.0 (reference)	1.2 (0.6, 2.3)	13.1 (6.3, 27.5)	<0.001	

*Adjusted birth weight, gestational age, small for gestational age, mechanical ventilation in the first week, PDA at day 7, creatine level in plasma at day 7, and hypertension during pregnancy*.

**Figure 1:**
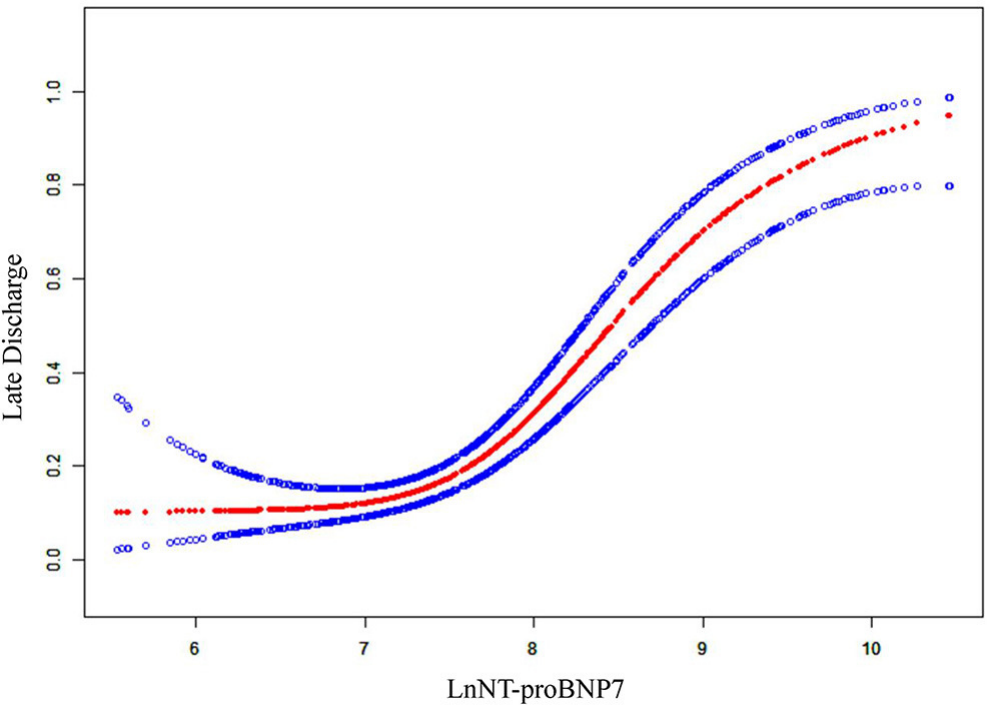
The relationship between LnNT-proBNP7 and late discharge. A non-linear relationship (S-shaped relationship) between them was detected after adjusting birth weight, gestational age, small for gestational age, mechanical ventilation in the first week, PDA at day 7, creatine level in plasma at day 7, and hypertension during pregnancy.

A similar dose–response relationship was observed in curve fitting analysis. Adjusted smoothed plots suggest a non-linear relationship (S-shaped relationship) between LnNT-prooBNP7 and late discharge (Figure 1). Using a two-piecewise linear regression model, we calculated the inflection points as 7.5 and 9. When LnNT-proBNP7 was below the first turning point (LnNT-proBNP7 = 7.5), late discharge was not associated with LnNT-proBNP7 (OR = 0.6; 95% CI, 0.2–1.6; *p* = 0.333). When LnNT-proBNP7 was above the first turning point but below the second turning point (LnNT-proBNP7 = 9), the risk of late discharge increased with the increase of LnNT-proBNP7 (OR = 5.0; 95% CI, 2.4–10.6; *p* < 0.001). When LnNT-proBNP7 was above the second turning point, the risk of late discharge did not increase with the increase of LnNT-proBNP7 (OR = 1.1; 95% CI = 0.2–6.1; *p* = 0.888) (Table 3).

**Table 3:** The results of two-piecewise linear regression model.

	**Crude β/OR (95% CI)*p*-value**	**Adjusted β/OR (95% CI)*p*-value**
**LnNT-proBNP7**		
<7.5	0.9 (0.4, 2.2) 0859	0.6 (0.2, 1.6) 0.331
≥7.5, <9	3.3 (1.8, 6.1) <0.001	5.0 (2.4, 10.6) <0.001
≥9	1.2 (0.3, 4.1) 0.789	1.1 (0.2, 6.1) 0.888

*Crude model: we adjusted sex*.

*Adjusted model: we adjusted birth weight, gestational age, small for gestational age, mechanical ventilation in the first week, PDA at day 7, creatine level in plasma at day 7, and hypertension during pregnancy*.

### Prevalence of Major Neonatal Morbidities and All Costs During Hospitalization of Infants in Different LnNT-proBNP7 Groups

Late discharge of preterm infants may result in higher costs during hospitalization and could be caused by following the major neonatal morbidities: BPD, NEC, and nosocomial infection. According to the turning points in the curve, we divided the infants into three groups and compared the costs during hospitalization and the prevalence of major morbidities between the three groups.

**Figure 2:**
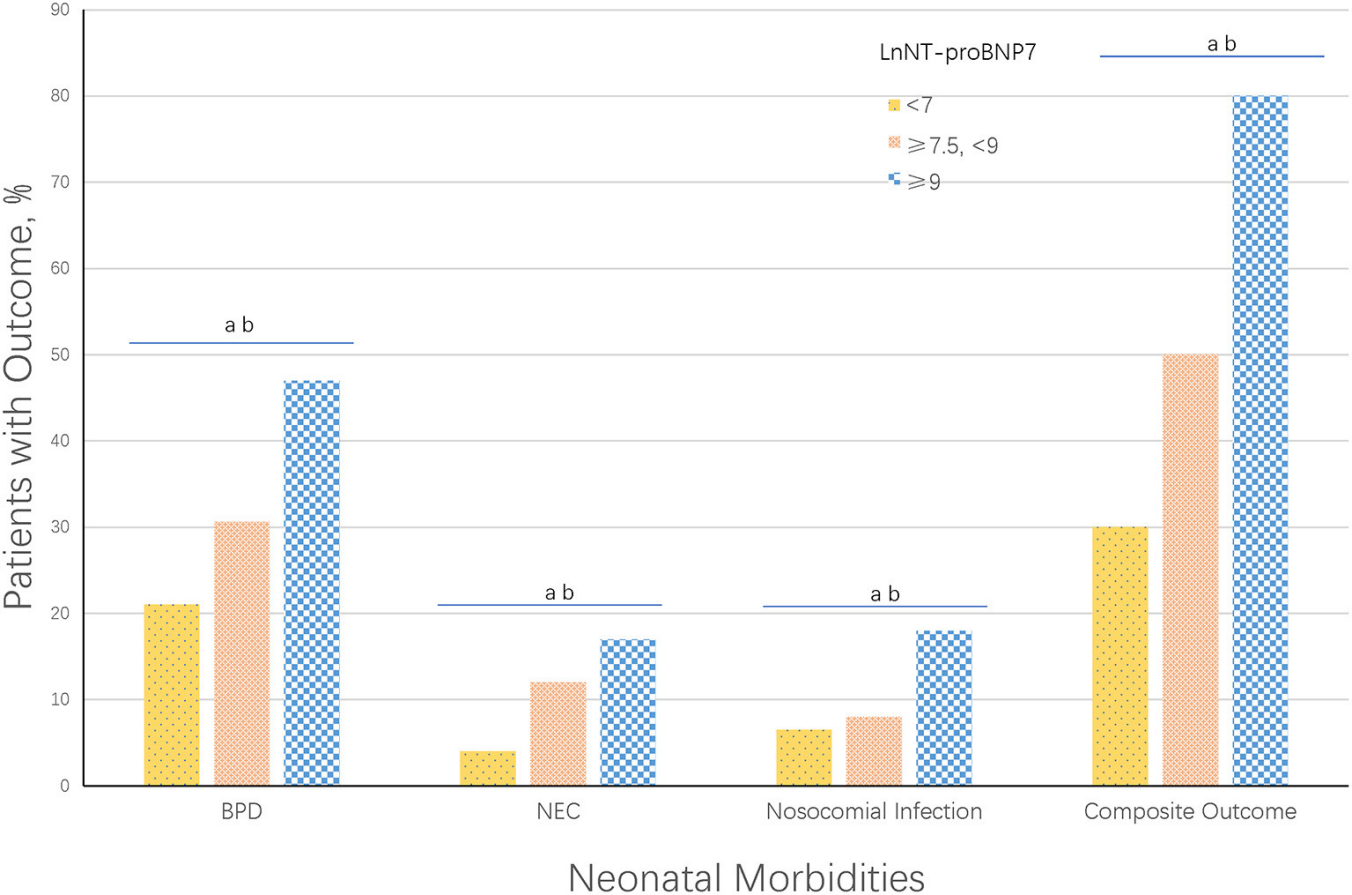
Neonatal morbidities by LnNT-proBNP7. Composite Outcome was defined as any of the following major neonatal morbidities: BPD, NEC, or nosocomial infection. ^a^*p* < 0.001, classical Levene's test comparing all 3 groups. ^b^*p* < 0.001, trend test comparing all 3 groups.

As shown in Figure 2, the prevalence of BPD, NEC (occurring after the first week), nosocomial infection, and any of the morbidities was highest in infants with LnNT-proBNP7 ≥ 9 and lowest in infants with LnNT-proBNP7 < 7.5, and the results of classical Levene's test and trend test showed that the difference was statistically significant (all *p* < 0.001). In addition, mean costs during hospitalization decreased with increasing GA. Among infants of the same GA, mean costs during hospitalization increased with increasing LnNT-proBNP7 (Supplementary Figure 2).

## Discussion

In this study of preterm infants born between 28 and 31 weeks, we found that the risk of late discharge increased according to increasing LnNT-proBNP7 in different subgroups. On further analysis, we found that the relationship between late discharge and LnNT-proBNP7 is not linear, and the S-shaped association in this study was independent of GA, birth weight, SGA, HSPDA at day 7, creatine level in plasma at day 7, and hypertension during pregnancy. To our knowledge, this study is the first study to show an association between length of stay in hospital and NT-proBNP level in preterm infants. Evidence indicates that elevated NT-proBNP levels are associated with impaired cardiac function (12), so cardiac function and the diseases affecting cardiac might represent factors that affect the length of hospitalization. However, it is still not clear whether heart function itself or a disease that affects heart function could better explain the association between higher NT-proBNP level and PLS.

Based on our data, most infants of 28–29 weeks' gestation were discharged home before 37 weeks' postmenstrual age and most infants of 30–31 weeks' gestation were discharged home before 36 week' postmenstrual age, which is consistent with two recent studies in the UK (26, 27). For infants with a long duration in the hospital, the difference in precise days in hospital is significant (28). Considering these concerns, we transformed days in hospital, a continuous variable, into late discharge, a categorical variable for analysis, and the statistical method was similar to a recent study performed by Hintz et al. (27).

The overall NT-proBNP levels in the plasma of term and preterm infants decreased rapidly during the first week of life (29), which is consistent with our observations. Despite this, during the first week of life, large fluctuations in NT-proBNP levels were observed when the preterm infants' illness or conditions such as deterioration of infection, frequent apnea, and feeding intolerance appeared, suggesting that the level of plasma NT-proBNP levels may be associated with the development of the condition or illness. However, the mechanism for the association between LnNT-proBNP7 and late discharge is not clear. Alicia et al. found that serum NT-proBNP levels during 48–72 h of life were associated with NEC (20). Our previous studies have also found that in Chinese infants, serum NT-proBNP in the first week may be an important predictor of severe ROP and BPD (30, 31). Prolonged mechanical ventilation and repeated intubation/extubation are important risk factors for BPD, and we also found serum NT-proBNP level could precisely predicted weaning failure of infants with RDS (ROC-AUC: 0.977; 95% CI: 0.918–0.997; *p* < 0.001) (18). These findings confirmed that a change in NT-proBNP level was associated with development of the condition. Therefore, in this study, we divided the infants into three groups by turning points of the smooth curve and compared the prevalence of NEC, BPD, and nosocomial infection among the three groups. The results indicated that the prevalence of NEC, BPD, nosocomial infection, or any disease morbidities was highest in the group with the highest LnNT-proBNP7 and lowest in the group with the lowest LnNT-proBNP7. To some extent, these findings explained the mechanism for the association between LnNT-proBNP7 and late discharge because the abovementioned diseases were important causes of late discharge.

Inherent factors including GA, BW, and maternal factors could simply estimate the discharge time of preterm infants. However, if it was not combined with the factors of the latter period of hospitalization, the estimation of the length of hospital stay was not very accurate (22, 23, 32). Therefore, it is important to find other early factors that could be potential to estimate the length of hospitalization. In our previous study, we used NT-proBNP to predict BPD and ventilator weaning (30, 31), which may influence the length of stay in hospital of preterm infants. In this study, we further found the relationship between NT-proBNP level and length of hospital stay, and it was meaningful to further explore the early predictors of the length of hospitalization of preterm infants.

We routinely tested the serum NT-proBNP levels of infants on the 1st, 3rd, 7th, and 14th days after birth, and tests of serum creatine levels and cardiac ultrasound were performed. However, we finally chose the NT-proBNP level on the 7th day as the exposure factor after statistical analysis and consideration. First, no significant correlation was noted between NT-proBNP level on the 1st day and late discharge (see Supplementary Material). Next, although the NT-proBNP level on the 3rd day was linearly correlated with late discharge (see Supplementary Material), there was a higher effect value between NT-proBNP level on the 7th day and late discharge, and the curve fitting suggested that a safe range of NT-proBNP levels potentially exists on the 7th day, which might be more valuable for clinical work. Then, one of the purposes of this study was to find potential indicators for the early prediction of length of hospital stay among preterm infants, so we did not take into account the NT-proBNP level on the 14th day. In addition, our previous research found the role of NT-proBNP levels on the 7th day in predicting BPD and the severity of BPD (30), so we planned to further explore the relationship between NT-proBNP level on the 7th day and PLS, which might lay a foundation for future studies on the NT-proBNP level on 7th day as an early predictor of length of stay.

The strengths of this study include that critical data were approximately complete with few losses. In addition, the non-linear relationship between LnNT-proBNP7 and late discharge and the result of the threshold effect analysis indicated that LnNT-proBNP7 below 7.5 (corresponding NT-proBNP level: 1,800 ng/ml) may be a “safe range” for very preterm infants, but prospective studies are needed to confirm this finding.

Some limitations to this study should be noted. First, this study is a single-center retrospective study; thus, we could not exclude potential referral bias. In our center, management for preterm infants was homogeneous, reducing the effect of different diagnosis and treatment methods on length of hospitalization stay. Second, the study population included Chinese preterm infants born between 28 and 31 gestation ages; therefore, the results may not apply to other ethnic groups and infants born at other GAs. Third, infants who died were not included in this analysis, which may influence our results although the number of dead infants (*n* = 10, of which 4 preterm infants survived for more than 7 days) was small. However, if death during hospitalization was treated equally with late discharge in analysis, our results may be more significant given that the NT proBNP levels of 4 dead preterm infants who survived for more than 7 days were very high (>10,000 ng/ml). Fourth, the population of this study did not include preterm infants transferred from other hospitals, so our results may not be applicable to these patients.

## Conclusion

Among preterm infants born at 28–31 weeks' GA, higher NT-proBNP7 levels were associated with longer hospitalization. The relationship between LnNT-proBNP7 and late discharge was S-shaped. LnNT-proBNP7 was positively related with late discharge when LnNT-proBNP7 was between 7.5 and 9.

## Data Availability Statement

The original contributions presented in the study are included in the article/Supplementary Material, further inquiries can be directed to the corresponding author/s.

## Ethics Statement

The studies involving human participants were reviewed and approved by Respective Research Ethics Boards of the First Affiliated Hospital of Zhengzhou University. Written informed consent to participate in this study was provided by the participants' legal guardian/next of kin.

## Author Contributions

JG and CL conceptualized and designed the study and drafted the initial manuscript. QZ and ML conceptualized and designed the study and reviewed and revised the manuscript. ZS, XC, WC, MZ, YZ, MS, WD, MW, SH, QX, HQ, and JW collected the data, carried out the initial analyses, and reviewed and revised the manuscript. FM and LW conceptualized and designed the study, coordinated and supervised the data collection, and critically reviewed the manuscript for important intellectual content. All authors approved the final manuscript as submitted and agree to be accountable for all aspects of the work.

## Funding

This study was supported by the National Health Commission Medical and Health Science and Technology Development Center, No. VA2020HK41, Provincial and Ministerial Co-construction Project, No. SBGJ2018040, Science and Technology Department of Henan Province Project, No. 172102410017, and Overseas Research and Training Project of Health Science and Technology Talents of Henan Province, No. HWYX2019066.

## Conflict of Interest

The authors declare that the research was conducted in the absence of any commercial or financial relationships that could be construed as a potential conflict of interest.

## Publisher's Note

All claims expressed in this article are solely those of the authors and do not necessarily represent those of their affiliated organizations, or those of the publisher, the editors and the reviewers. Any product that may be evaluated in this article, or claim that may be made by its manufacturer, is not guaranteed or endorsed by the publisher.
