# Supplementary material for: Association Between NT-proBNP and Prolonged Length of Stay in Hospital Among Preterm Infants Born at 28–31 Weeks' Gestation
Source: Front Pediatr. 2022 Jan 24;9:783900. doi: 10.3389/fped.2021.783900 (PMC8819076; doi:10.3389/fped.2021.783900)
Supplement: Supplementary file 2 [file Data_Sheet_1.docx]

**Supplementary Appendix**

This appendix has been provided by the authors to give readers additional information about their work.

**Supplementary Web Appendix**

**Association Between NT-proBNP and Prolonged Length of Stay in Hospital Among Preterm Infants Born at 28 to 31 Weeks' Gestation**

| Table of Contents | Page |
| --- | --- |
| TableS1. PMA in Days at Discharge of the Fourth Quartile According to EGA Group | 2 |
| TableS2. Association of Infant and Maternal Characteristics with LnNT-proBNP7 (Univariate Analysis). | 2 |
| TableS3. Association of Infant and Maternal Characteristics with Late Discharge (Univariate Analysis). | 2 |
| FigureS1: Flow Diagram of the Study Cohort | 3 |
| FigureS2. Costs During Hospitalization by LnNT-proBNP7 | 4 |
| Supplementary Material for Discussion Part | 4 |

**eTable 1. PMA in Days at Discharge of the Fourth Quartile According to EGA Group**

| Fourth Quartile | PMA, d | N (% in EGA Group) |
| --- | --- | --- |
| 28 wk EGA | ≥264 | 22(26) |
| 29 wk EGA | ≥265 | 41(26) |
| 30 wk EGA | ≥260 | 51(26) |
| 31 wk EGA | ≥259 | 72(25) |

Abbreviations: PMA, postmenstrual age; EGA, estimated gestational age; wk, week

**eTable 2. Association of Infant and Maternal Characteristics with LnNT-proBNP7 (Univariate Analysis).**

| Characteristics | Outcome: LnNT-proBNP7 | | |
| --- | --- | --- | --- |
|  | β | 95%CI | P Value |
| Gestational age |  |  |  |
| <29 | Reference |  |  |
| >=29, <30 | -0.3 | (-0.5, -0.1) | .017 |
| >=30, <31 | -0.5 | (-0.7, -0.2) | <.001 |
| >=31 | -0.6 | (-0.8, -0.4) | <.001 |
| Birth weight | -0.0 | (-0.0, -0.0) | <.001 |
| SGA (yes vs no) | 0.3 | (0.1, 0.4) | <.001 |
| Creatine level at day 7 | 0.0 | (0.0, 0.0) | <.001 |
| HSPDA at day7 (yes vs no) | 1.1 | (0.8, 1.3) | <.001 |
| Mechanical ventilation in the first week (yes vs no) | 0.8 | (0.6, 0.9) | <.001 |

**eTable 3. Association of Infant and Maternal Characteristics with Late Discharge (Univariate Analysis).**

| Characteristics | Outcome: Late Discharge | | |
| --- | --- | --- | --- |
|  | β | 95%CI | P Value |
| Birth weight | 1.0 | (1.0, 1.0) | <.001 |
| SGA (yes vs no) | 6.3 | (4.3, 9.2) | <.001 |
| Mechanical ventilation in the first week (yes vs no) | 2.5 | (1.8, 3.6) | <.001 |
| Hypertension during pregnancy (yes vs no) | 1.9 | (1.4, 2.7) | <.001 |
| NEC in the first week (yes vs no) | 3.9 | (1.6, 9.4) | .002 |
| LnNT-proBNP7 | 3.2 | (2.5, 4.0) | <.001 |

**eFigure 1: Flow Diagram of the Study Cohort**


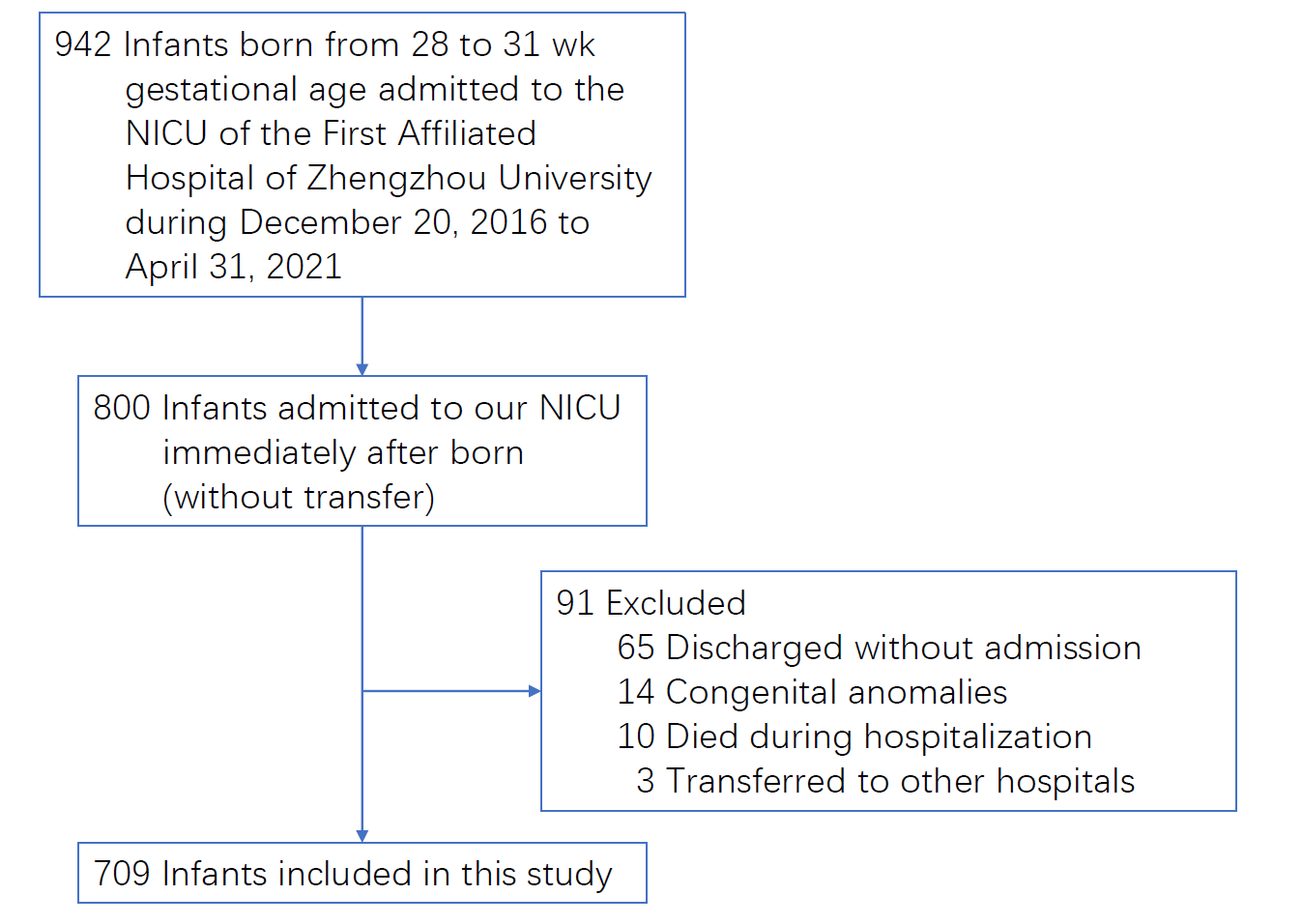


**eFigure 2. Costs During Hospitalization by LnNT-proBNP7**


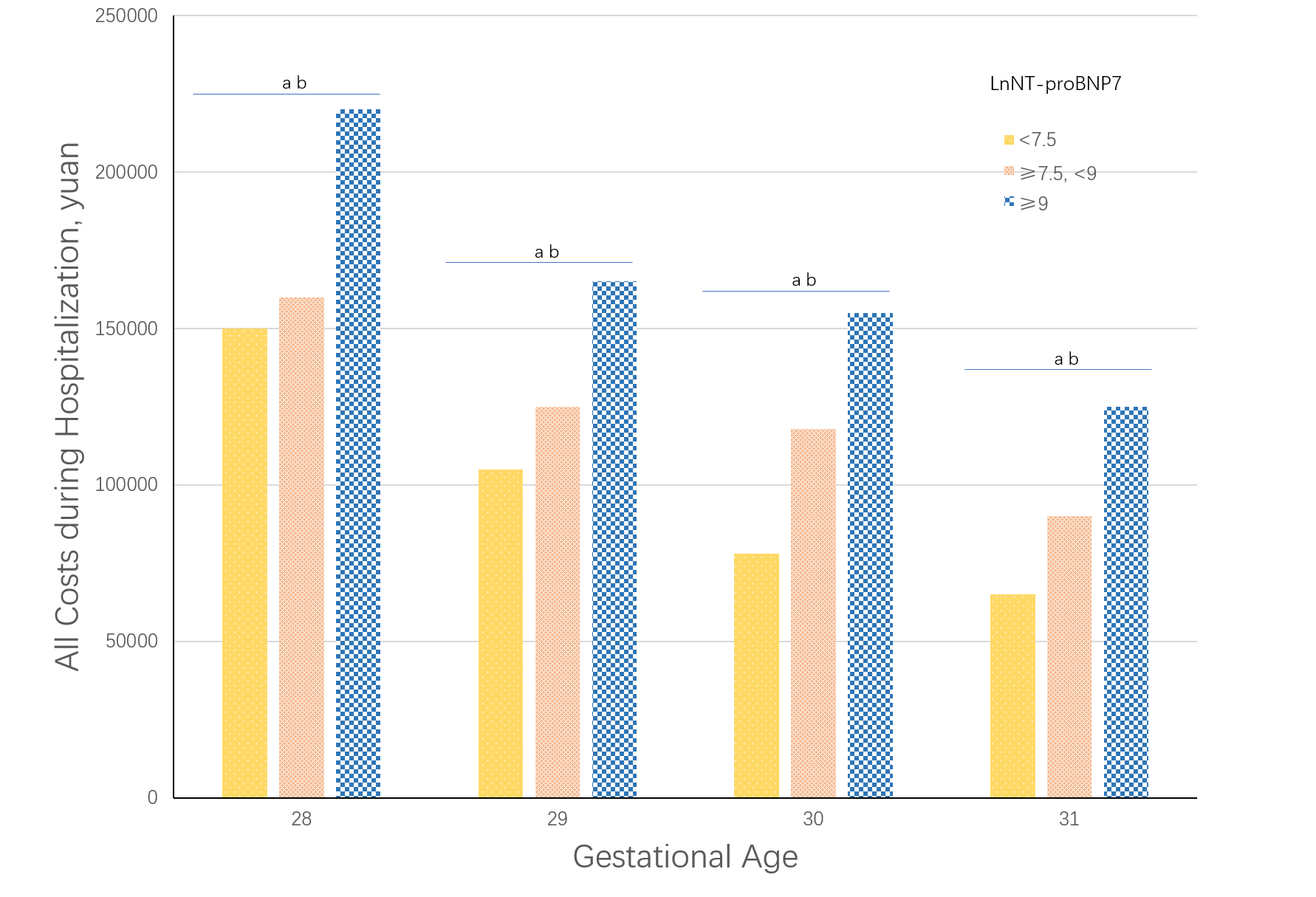


^a^ *P* < .001, Classical Levene's test comparing all 3 groups

^b^ *P* < .001, ltrend test comparing all 3 groups

**Supplementary Material for Discussion Part:**

**The Relationship Between LnNT-proBNP1, LnNT-proBNP3 and Late Discharge**

| Characteristics | Outcome: Late Discharge | | |
| --- | --- | --- | --- |
|  | OR | 95%CI | P Value |
| LnNT-proBNP1 | 1.0 | (0.9, 1.1) | 0.964 |
| LnNT-proBNP3 | 1.6 | (1.3, 2.0) | <.001 |

Adjusted birth weight, gestational age, small for gestational age, and hypertension during pregnancy.

CI, confidence interval.

**The Relationship Between LnNT-proBNP1, LnNT-proBNP3 and Late Discharge**


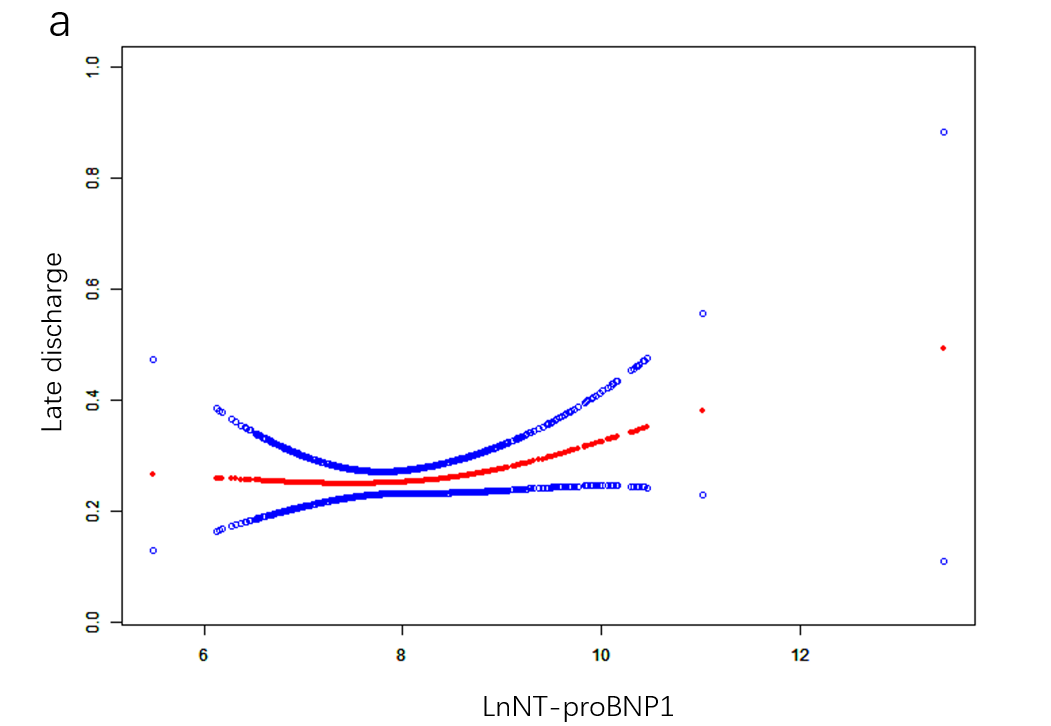


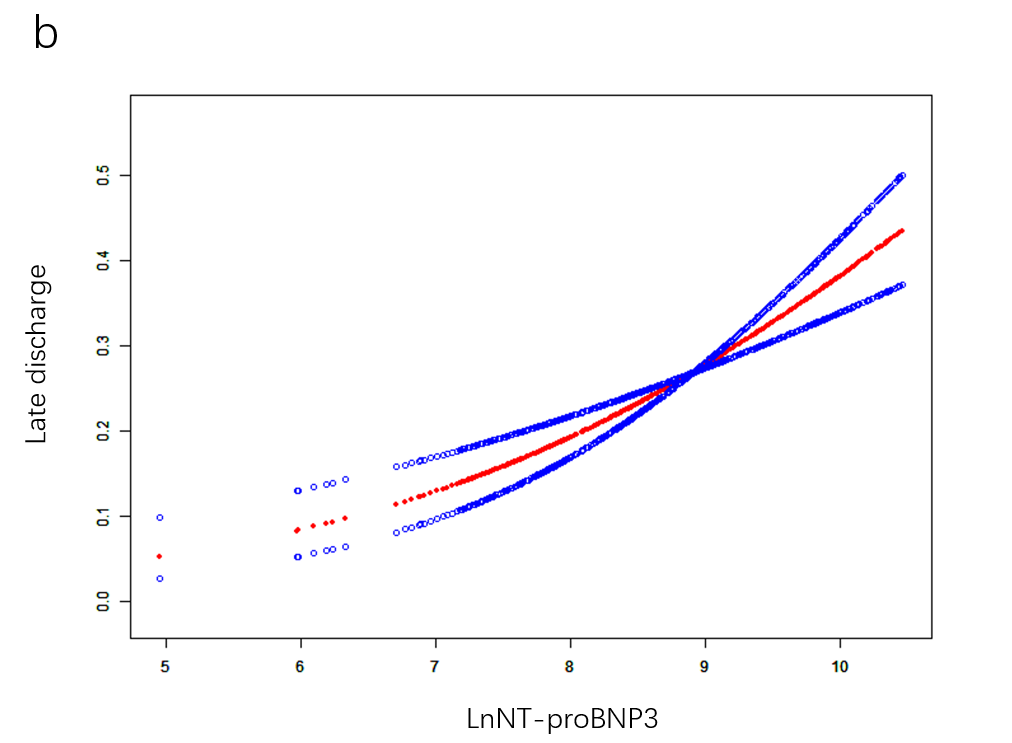


1. Smooth curve of the relationship between LnNT-proBNP1 and late discharge
2. Smooth curve of the relationship between LnNT-proBNP3 and late discharge

Adjusted birth weight, gestational age, small for gestational age, and hypertension during pregnancy.

Abbreviations: LnNT-proBNP1, logarithm of NT-proBNP level on the 1st day; LnNT-proBNP3, logarithm of NT-proBNP level on the 3rd day
